# Hong Kong anti-human trafficking framework: what lessons can be learned from Europe?

**DOI:** 10.3389/fsoc.2024.1395907

**Published:** 2024-09-11

**Authors:** Noble Po-kan Lo

**Affiliations:** ^1^College of Professional and Continuing Education, The Hong Kong Polytechnic University, Hong Kong, Hong Kong SAR, China; ^2^Department of Educational Research, Faculty of Arts and Social Sciences, Lancaster University, Lancaster, United Kingdom

**Keywords:** human trafficking; anti-human trafficking framework, Hong Kong’s Modern Slavery Bill, Council of Europe, European Convention on Human Rights, European Court of Human Rights

## Abstract

This article examines the regulatory framework related to human trafficking in Hong Kong and identifies its deficiencies as the lack of an accepted internationally compliant definition of trafficking and the absence of any specific criminal offence of trafficking as a result. The article compares the approach taken in Hong Kong to efforts undertaken in Europe by the Council of Europe, the UK, and the European Union and identifies several lessons from the European experience that could help rectify failures observed in Hong Kong. In particular, effective combatting of human trafficking requires not only a definition of the offence that recognises the essential elements—an “act,” a “means,” and a “purpose” of exploitation—but also the establishment of sufficient state institutions and agencies dedicated to identifying and protecting trafficking victims. Without the detection of trafficking victims, criminals engaged in the act of trafficking perceive their chances of being caught and prosecuted as low and operate with impunity. This necessitates the adoption of a consistent and readily identifiable criminal offence of “trafficking” aligned with the approach taken by the Palermo Protocol, just as the EU and UK have done.

## 1 Introduction

Human trafficking is an extremely serious issue. The European Parliament (2005) has described trafficking as being one of the main forms of organised crime experienced around the world today. Its impact on those trafficked, their loved ones, and their families, as well as on societies blighted by other forms of criminal activity supported by and generated by organised crime, is immense. Indeed, the International Labour Organization (ILO) estimated in 2012 that there were some 21 million people trafficked in the world at large (International Labour Organization, 2012). The research goal of this article is to determine the adequacy of Hong Kong’s anti-human trafficking framework and to identify what lessons, if any, can be learned by Hong Kong from jurisdictions in Europe (in particular the EU) and the non-EU state (the UK).

The structure of this article will be as follows: The next section will examine in more detail the deficiencies of the law in Hong Kong. Once the key deficiencies of the law are identified, the following section will consider what lessons can be learned by Hong Kong legislators from the law as it stands in the UK and the EU in Directive 2011/36/EU and other relevant legislative provisions such as those of the Council of Europe and its anti-trafficking convention. Furthermore, a comparative assessment of the way in which the Hong Kong Court of Final Appeal in the case of *ZN Secretary for Justice and Others* [2019] HKCFA 53 and the European Court of Human Rights have, respectively, interpreted the obligation on states to positively take steps to prevent “trafficking” will be examined; this is of interest as neither the International Covenant on Civil and Political Rights 1966 (International Covenant on Civil and Political Rights 1966) to which Hong Kong is a signatory nor the European Convention on Human Rights 1950, which the EU and its Member States are signatories to, contains a specific express right against being “trafficked.” Both courts, however, have come to different conclusions on what obligation the state has to prevent such acts.

### 1.1 Methodology

This study hypothesises that the law relating to anti-human trafficking in Hong Kong is inadequate to deal with the seriousness of the problem and the wide range of means or mechanisms by which trafficking might be carried out. It will be further suggested that there is scope for the legislature in Hong Kong to identify lessons and adopt models that have been more successfully used elsewhere in Europe, for example. The end goal of such a piece of research is to recommend a more efficacious model of fighting human trafficking that Hong Kong could adopt. This might thereby contribute to reducing the scope for Hong Kong to be used as a base for human trafficking and forced labour operations in the Asia-Pacific region. This is something that is of increasing concern in the region, as demand for foreign labour in Hong Kong from neighbouring states such as the Philippines or Indonesia and the potential for coerced trafficking are not necessarily for the purposes of prostitution or other forms of sexual exploitation alone.

To determine, however, whether this hypothesis is accurate, it is necessary, first of all, to be able to identify the position of the law in Hong Kong as it relates to anti-human trafficking. The methodological approach best suited to identifying the specific provisions of law as they relate to a given issue is the doctrinal model (Ishwara Bhat, 2020). The doctrinal, or black letter model, is a legal methodology aimed at identifying the relevant sources of law in a given area and synthesising these around a range of facts or scenarios so that the precise and accurate position of law as it applies to a given question can be ascertained. Here, this question is, what does the law in Hong Kong identify as “human trafficking?” Gauging the efficaciousness or quality of this law, however, as a means of combatting human trafficking in a legal vacuum without reference to accepted international definitions of trafficking, and without reference to other comparative approaches that have been taken by other states to the same (or similar) problems is difficult. Without any objective basis for comparison, it might be that the law, as it stands, is simply better than nothing, but it would be difficult to suggest that the law is as good as it can be in preventing trafficking from taking place. Given that there is, however, an international definition of human trafficking which the international community has sought to introduce in the Palermo Protocol to attempt to enhance and co-ordinate cross-border anti-human trafficking efforts, there is in fact some degree of basis for a comparative methodological approach to be taken here. From a functionalist approach, the law in Hong Kong can then be compared to the way in which other states around the world have sought to combat human trafficking. Specifically, this will be pursued through an analytical comparison of the definition of “trafficking” utilised in Hong Kong’s law, as well as any other associated and relevant legal provisions, such as the domestic Hong Kong law in Article 4 BOR, which prohibits *inter alia* forced labour, to that set out in the Palermo Protocol itself, and the approach taken by the UK and the EU more generally.

### 1.2 What is trafficking?

Before going any further, the obvious question arises here as to what is meant by the term “trafficking.” As will be seen, definitional issues plague this area, and even a concept as important as the trafficking of human beings does not yet enjoy a universally accepted and non-contentious definition (Jansson, 2015, p. 2). The failure to understand and subsequently draft effective anti-trafficking legislation is a serious problem facing international and national crime agencies. This is because regulatory differences may allow traffickers to capitalise on legislative gaps, divergences, and loopholes that might exist between one state and another (Lo, 2024a). The act of trafficking is typically regarded as being one that involves some form of movement of persons (although, as will be seen, this is not necessary under some definitions of trafficking), either inside or outside of a jurisdiction, but the movement of persons and their exploitation tend to take place more readily when vulnerable people are removed from their homes, families, and communities. When these trafficked people are moved abroad to a state in which the authorities have no data or information about these people or their origins, and in which no friends or family exist, identifying and rescuing such individuals or bringing their traffickers to justice, may be made much more difficult than might otherwise be the case.

Human trafficking is, in essence, an international problem. International responses include conventions, such as the Palermo Protocol (whose full title is the Protocol to Prevent, Suppress, and Punish Trafficking in Persons, Especially Women and Children, supplementing the United Nations Convention on Transnational Organized Crime), which, amongst other things, seeks to provide an international definition of trafficking (Office of the United Nations High Commissioner for Human Rights (OHCHR), 2004). This definition is set out in Article 3(a) of the Palermo Protocol, which provides that trafficking in persons means:

“The recruitment, transportation, transfer, harbouring or receipt of persons, by means of threat or use of force or other forms of coercion, of abduction, of fraud, of deception, of the abuse of power or of a position of vulnerability or of the giving or receiving of payments or benefits to achieve the consent of a person having control over another person for the purposes of exploitation.”

In addition to this, Article 3(a) of the Palermo Protocol goes on to provide a definition of exploitation, including:

“At a minimum, the exploitation of the prostitution of others or other forms of sexual exploitation, forced labour or services, slavery or practices similar to slavery, servitude, or the removal of organs.”

It is established by Article 3(b) that even where a person has ostensibly consented to having been “trafficked,” such consent is irrelevant for the purposes of determining whether trafficking has indeed taken place where any of the means, or processes, as they might be regarded as being, set out in Article 3(a), such as force, threat of force, coercion, abduction, fraud, deception, and so on, are used. As was noted earlier, it is not necessarily essential that a movement of a person takes place according to this definition for trafficking to occur; the mere harbouring of a person by another with the requisite “means” and for the “purposes” of exploitation is instead sufficient.

In short, it might be said that there are three key elements to “trafficking” recognised by the Palermo Protocol. The protocol recognises that the essential elements of trafficking occur on the basis of an “act,” a “means,” and a purpose. Thus, there is an “act,” which occurs such as through recruitment, transportation, harbouring, or receipt of a person by another. There is subsequently a requirement for some means, such as force, threat, coercion, or deception. Finally, the “purpose” or end goal must be the exploitation of the person, and whether this is for their sexual exploitation, labour, services, servitude, or indeed their bodily organs, is irrelevant for these purposes. These three elements of trafficking can be concisely regarded as being the act, the means, and the exploitative purpose; the act is the harbouring, receipt, and transfer, the means being through the use of force, threat, or coercion, and the third being the intent to exploit the other.

Establishing a uniform and acceptable definition of trafficking is important to avoid legislative gaps and divergences being exploited by traffickers (Lo, 2024b). Some states have historically regarded the “transportation” or “transfer” element as important, whilst others have focussed on the fraud, force, or coercion of others with a view to their exploitation. Some, such as Hong Kong (which is not a signatory to the Palermo Protocol), require as part of their own domestic law on trafficking a requirement that a person be exploited sexually before they can be recognised as being trafficked. It is this definitional difficulty that will be studied and analysed in this article.

### 1.3 Background and context

As noted above, Hong Kong is not a signatory to the Palermo Protocol, and its anti-trafficking framework is not conformant to the Protocol. Instead, section 129 of the Hong Kong Crimes Ordinance (Cap 200) provides only that a person commits an offence if they “take part” in the bringing of another person into Hong Kong or in the taking of another person out of the territory of Hong Kong, “for the purpose of prostitution.” Nothing is said in section 129 of the Crimes Ordinance that there is a need for such a form of trafficking to be based upon force, threat, coercion, abduction, fraud, deception, or the abuse of power or vulnerability of the trafficked person, and there is nothing said as to the requirement for another person to be otherwise capable of being controlled other than for sexual purposes. Nor is it possible under the law in Hong Kong, at least *prima facie*, under s129 Crimes Ordinance, for a person to be considered “trafficked” if they are moved, held, or controlled by another person internally within Hong Kong itself, such as by the internal “harbouring” or “receipt” of persons with a view to their exploitation, even if they are held as such for the purposes of prostitution. This is because in such a purely domestic scenario, there would be no element satisfying the requirement that a person be moved “in” or “out” of Hong Kong as required by law. Nor would it be the case that a person could be legally considered “trafficked” under the law in Hong Kong as set out in s129 Crimes Ordinance if the purpose of that trafficking was not their sexual exploitation in the form of prostitution but for some other reason, such as for their use as forced labour, domestic servitude, or what might be termed “slavery” (or modern slavery) (Harrison, 2020, p. 19).

The legal framework related to human trafficking in Hong Kong appears, therefore, to be inadequate and incapable of properly combatting human trafficking. There are many critics who share such concerns. Chan (2014), for example, suggests that the law in Hong Kong is failing many victims who are being forced or exploited in Hong Kong outside of a sexual context. For this author, recent cases before the courts of Hong Kong in which domestic “helpers” have been abused have raised the spectre of potentially large amounts of people being held in Hong Kong in positions akin to slavery. The limited scope of Hong Kong’s regulatory framework with respect to human trafficking is also identified by Anderson and Li (2018), for example, who write that the great difficulty with the law in Hong Kong is, as suggested above, definitional. In particular, the problem that has already been identified in this article is that the legislative framework in Hong Kong only defines human trafficking as being capable of being committed in the context of cross-border sex work. These authors in particular consider the position of refugees in Hong Kong, who are at risk of suffering from human trafficking, at least when defined under Article 3 of the Palermo Protocol. It is difficult to identify these individuals and to protect them from harm. This is because the authorities, in line with the legal framework, regard refugee status and the risks of refoulment, for example, and trafficking as being entirely separate issues. The US State Department, meanwhile, in its assessment of the legal position in Hong Kong, suggests that the law in Hong Kong is presently unable to “meet the minimum standards” for the “elimination of trafficking” and has recommended that the legislature, amongst other things, introduce specific legislation to criminalise trafficking of human beings in accordance with the definition set out in the Palermo Protocol (United States State Department, 2022, 2023).

For the background of the study, it is hypothesised that the law in Hong Kong with regard to human trafficking remains, at the time of writing, inadequate as it fails to properly define “human trafficking” in the way in which the Palermo Protocol and its signatory states do. This allows too many forms of exploitation which might take place in Hong Kong and in a cross-border context to take place without those engaging in or assisting in this form of activity being legally liable as “traffickers.” Hong Kong is not a signatory to the Palermo Protocol. The Government has argued that it will not sign nor ratify the agreement because it has concerns that the other obligations in the Protocol, such as those under Article 6(3)(a) to provide “appropriate housing,” for example, to victims of trafficking, might be abused by migrants seeking to claim state support in Hong Kong despite not being trafficking victims (Huang, 2021). Additionally, as Huang reports, the Government suggests that Hong Kong domestic law in the Bill of Rights Ordinance (BOR) Cap 383 already sufficiently protects individuals from “trafficking” under the Palermo Protocol’s definition because it prohibits forced labour or servitude.

However, as will be shown, this is insufficient to properly prevent and protect against a wider definition of “trafficking.” The BOR, itself based on the International Covenant for Civil and Political Rights of 1966 (which prohibits “servitude” or forced labour in Article 8), does not apply to the processes which are an inherent part of the trafficking crime as defined in Article 3 of the Palermo Protocol (processes such as transfer, receipt, or holding of another person), and there remains concern therefore that the law in Hong Kong is improperly situated to identify and prosecute trafficking as so defined. This article will suggest that Hong Kong ought to introduce a specific anti-human trafficking law which complies with the Palermo Protocol’s definition of “trafficking.” To ascertain the basis on which such a law ought to be modelled, this article will analyse, from a comparative perspective, the way in which some jurisdictions in Europe have addressed human trafficking in their own law. These states, such as the UK, and those within the European Union (EU), do have domestic law which conforms more clearly to the Palermo Protocol’s provisions. Indeed, within the EU, Member States are obliged to have transposed the EU’s Directive 2011/36/EU on Preventing and Punishing in Human Beings, a directive based directly on the Palermo Protocol’s provisions. What lessons Hong Kong and its legislature might glean from these European legislative efforts will be examined. The position of EU law, as well as the position of UK law, have been selected for the purpose of comparative research because these jurisdictions are examples of the way in which legislatures might take steps to prevent and punish “trafficking” in a wider sense than that pursued by the law in Hong Kong; the UK has been selected because of the similarity of its legal system to that of Hong Kong.

## 2 The anti-human trafficking regulatory framework of Hong Kong

### 2.1 Section 129 of the Crimes Ordinance (Cap 200)

As noted in the introduction, the starting point for this study is to analyse the position of the law in Hong Kong as it pertains to anti-human trafficking regulations. There are a number of legislative provisions which are relevant; it is not only the case that the provisions of the s129 Crimes Ordinance might be regarded as regulating activities which the Palermo Protocol terms “trafficking.” Additionally, the law in Hong Kong in instruments such as the BOR will be of importance here, as was seen in the Hong Kong Court of Final Appeal’s (HKCFAR) decision in *ZN Secretary for Justice and Others* [2019] HKCFA 53. This case and its implications for the law on human trafficking in Hong Kong will be examined in due course, and it will be suggested in this article that the decision of the court that there is no positive legal obligation on the legislature in Hong Kong created by Article 4 BOR to criminalise a wider definition of “trafficking” than that presently set out in Hong Kong law is unhelpful.

It is, however, in section 129 of the Crimes Ordinance that explicit reference to the crime of “trafficking” under the law of Hong Kong is made. As was noted in the introduction, section 129(1) of the Crimes Ordinance states that any person:

“Who takes part in bringing another person into, or taking a person out of, Hong Kong, for the purpose of prostitution, shall be guilty of an offence ….”

Section 129(2) of the same ordinance then goes on to explain that the consent of such a person to being trafficked either into, or out of Hong Kong is unable to create a defence here, whether the person in question knew or did not know, that they were being “trafficked” for the purposes of prostitution, or that they received any advantage (such as payment) for their exploitation as such.

To some degree, it is fair to say that the legislature of Hong Kong has indeed sought to at least try and prohibit “trafficking” in Hong Kong. However, even a cursory look at the definition of “trafficking” in s129(1) of the Crimes Ordinance allows one to determine that the scope of this prohibition is incredibly narrow when compared to the definition of “trafficking” set out in the Palermo Protocol itself. In the first place, whilst the Palermo Protocol in Article 3(a) explains that trafficking can be constituted by the “harbouring,” “receipt,” or “recruitment” of persons, as well as by their “transfer,” the law in s129(1) Crimes Ordinance refers only to the “transfer” element, indicating a requirement of cross-border movement of persons is necessary in order for the person alleged in Hong Kong to have engaged in “trafficking” to be guilty of the offence. This is not something which the key international instrument in the form of the Palermo Protocol requires and excludes a significant range of behaviours from the scope of the “trafficking” offence set out in s129(1) as a result.

This definitional lacuna identified in the law of Hong Kong appears to indicate that a purely domestic situation, in which a person may be recruited for the purposes of sexual exploitation or to engage in non-consensual work as a prostitute (or who consents under some coercion or deception to doing so), would not necessarily fall under the ambit of “trafficking” as defined in the law in Hong Kong itself. Whilst it is the case that other provisions of law do exist here to protect individuals against servitude and forced labour (notably those in Article 4 of the BOR, as will be seen), it is also the case, as Emerton et al. (2007) note, that the failure to identify these domestically trafficked (at least under the definition set out in the Palermo Protocol) individuals as being “trafficked” results in subsequent failures on the part of the authorities to identify these vulnerable individuals, and to offer them the support and protection which they might require. One of the requirements imposed by the Palermo Protocol in Article 6(3) placed on signatories is to ensure that the state considers the implementation of measures designed to provide for the “physical, psychological, and social recovery” of trafficking victims. This might include the necessity, amongst other things, to secure housing for them [specifically provided for under Article 6(3)(a) of the Protocol] or to provide counselling, medical assistance, or employment information as to the person’s rights (which they might have been deprived of by their traffickers) and educational and training opportunities for these persons.

The rationale for the Protocol’s emphasis on these matters is obvious: the nature of a trafficked person’s existence is marked by vulnerability, and by providing some of the basic needs of a person, such as medical treatment, safe housing, knowledge as to their rights and options, and training, education, or employment, we can contribute to reducing that individual’s personal level of vulnerability to further trafficking or exploitation in the future. Trafficking, as a crime, is an offence which preys on vulnerability and on the ignorance and lack of understanding of the legal rights enjoyed by persons to their own autonomy (Toney-Butler et al., 2023). Indeed, these obligations and the concerns that they might be used by unscrupulous individuals to obtain access to scarce public funds or housing available in Hong Kong were given in the *ZN* case as a reason for China’s continued reluctance to allow Hong Kong’s ratification of the Palermo Protocol. It is submitted that this highlights an implicit acknowledgement that the law in Hong Kong does not provide the same stringent degree of protection and rights for victims of trafficking that international law in the Protocol itself would be capable of offering.

The Palermo Protocol is arguably an effective instrument for harmonising and encouraging the combatting of trafficking around the world because it recognises that a person is capable of being trafficked by a number of means, through a number of different acts, all for the purpose of exploitation. This is in stark contrast to the position that the law in Hong Kong allows domestic victims of “trafficking” to escape effective identification and subsequent intervention by the state because of its sole focus on the transfer either into, or out of, Hong Kong itself. If, as was noted in the introduction, the Palermo Protocol’s definition of trafficking has three key elements (the act, means, and purpose), the failure of the law in Hong Kong to bring within its scope domestic control, harbouring, transfer, or receipt of a person fails to fulfil the first of these elements.

The second key definitional limitation exhibited by s129(1) of the Crimes Ordinance and its elements is the fact that in order to be convicted of a “trafficking” offence, the defendant must have sought to exploit the victim of trafficking solely for the purposes of prostitution or other forms of sexual exploitation. This is a legacy to some degree of the fact that the Crimes Ordinance, and s129(1) itself, were drafted and brought into force prior to the United Nations Convention on Transnational Organised Crime of 2000 being itself adopted. The law in Hong Kong in this respect retains much of the moralistic concerns over vice which the old colonial British law in force in Hong Kong during British rule displayed in that the key concern of the law here is arguably to prohibit and prevent prostitution from taking place, rather than to protect individuals from being forced or coerced into sex work (Lim, 2015, p. 92). In addition to this, however, the law here has also been strongly influenced by feminist legal theory and criticism, particularly through the works of radical feminist authors such as Jeffreys (1997, p. 345), who suggest that the very act of prostitution (whether consensual or not on the part of the sex worker) consists of a form of brutalisation, on the part of men, against women and their rights to bodily autonomy.

That such an attitude, combined with a legislature intent on cracking down on the perceived sinful vice activities and criminal organisations which typically operated brothels in Hong Kong and in the region in the past, resulted in the focus on trafficking laws being on “prostitution” rather than other forms of exploitation, is perhaps not surprising. It is, however, the case that the continued failure of the legislature to rectify such a position in light of the adoption by the United Nations General Assembly of the Palermo Protocol now calls into question the adequacy of the law in Hong Kong. In particular, it is made clear by Article 3(a) of the Palermo Protocol that the purposes for which a person might wish to exercise control over the trafficked individual in order for the act to constitute “trafficking” might be for a whole range of exploitative purposes. It is expressly provided that these purposes are not limited to and include only as a “minimum” the prostitution of others or their sexual exploitation. Thus, the failure to include in s129(1) any purposes other than prostitution for which another person’s movement is exercised to constitute trafficking, indicates that the Hong Kong law fails to fulfil the third key element of the Palermo Protocol’s definition, in the form of exploitation.

There is little doubt that this failure to recognise the receipt, harbouring, transfer, or holding of persons motivated by exploitation for purposes other than sexual exploitation alone causes difficulties in Hong Kong. As Chan (2014) writes, the two key sectors of the economy in which human beings are trafficked into Hong Kong are the prostitution sector and forced domestic labour. Having a trafficking prohibition which covers the first, but not the second of these is inexplicable, and renders the position of Hong Kong law inadequate and unable to deal with the modern problems caused by human trafficking into and within the region. As Leung et al. (2022) have suggested, these failures make it more difficult for the authorities to both identify victims of trafficking engaged in non-sexual forms of forced labour or other forms of exploitation and result in a subsequent failure to provide these individuals with the protection of the state. Traffickers who bring others into Hong Kong for the purposes of exploitation using force, coercion, or deception may do so largely without impunity as a result.

### 2.2 Article 4 Bill of Rights Ordinance (Cap 383)

Despite the protestations of critics, as noted above, who have argued that the Crimes Ordinance fails to adequately identify “trafficking,” there have historically been suggestions that there is no real need for the law to be amended in this area (Marwah and Ho, 2019). The reason for this, as suggested by Marwah and Ho (2019) article, is that the authorities have tended to suggest that Article 4 of the Hong Kong BOR already adequately prohibits other forms of exploitation such as domestic servitude or forced labour, or that exploitation and forced transplantation of human organs (another concern prevalent in Hong Kong and in the Asian region where there is something of a shortage of organs for medical uses) is already prohibited by rules in the Human Organ Transplant Ordinance (Cap 465), for example.

Article 4 of the Hong Kong BOR does indeed prohibit slavery and servitude. Under Article 4(1) “slavery” is prohibited in all its forms, whilst Article 4(2) prohibits “servitude.” Thus, both the owning of another human being and the coerced service of another are prohibited (Allain, 2015). Similarly, Article 4(3) prohibits forced or compulsory labour, with some exceptions such as for detained prisoners, during military service, or during times of public emergency, for example. The BOR and the provisions of Article 4 of the BOR are consistent with the rules of the International Convention for the Protection of Civil and Political Rights 1966 (ICCPR), which Hong Kong has ratified and which are incorporated into Hong Kong domestic law under Article 39 of the Hong Kong Basic Law itself. As Swepston (2014), writing for the ILO, explains forced labour has an accepted understanding both of the terms “slavery and servitude” and of the term “forced labour” here under international law; the term “slavery” is accepted as being that first defined and set out in a predecessor treaty internationally in the form of the Slavery Convention 1926, which defined slavery as being “the status or condition of a person over whom all or any of the powers attaching to the right of ownership are exercised.”

Subsequent treaties such as the Supplementary Convention on the Abolition of Slavery, the Slave Trade, and Institutions and Practices Similar to Slavery 1956 added “practices similar to slavery” within the scope of these term meanings. Thus, debt bondage, serfdom, freedom from forced marriage, and forced child labour were all prohibited as being forms of servitude under the Convention. Forced labour, meanwhile, is a concept which has been developed largely through the work of the ILO, through the first Forced Labour Convention drafted by the International Labour Organization (1930) [Forced Labour Convention 1930 (No. 29)]; the need for such a convention was regarded at the time as being necessitated by colonial practices whereby the state and its authorities compelled native workers to work, often for no reward, to help build infrastructure or other projects. Since there was not necessarily any private party who could be identified as an “owner” of such persons, a distinction had to be capable of being drawn between the concept of “slavery” and the understanding that the state or authorities belonging to the state ought also to be prohibited from exploitation of the forced or compelled labour.

Article 2 of this Convention thus defines “forced labour” without any reference to slavery as being “all work or service which is extracted from any person under the menace of any penalty and for which the said person has not offered himself voluntarily.” Minor amendments were subsequently made by the ILO in the Abolition of Forced Labour Convention of 1957, which sought to ensure that forced labour was not used by states as a form of punishment for holding or expressing certain political views, for example. This international background is included here to highlight the fact that the courts in Hong Kong have at their disposal a significant degree of background material, including *travaux preparatoires* for these treaties on which the domestic law in Hong Kong in the BOR prohibiting forced labour, slavery, and servitude is based.

### 2.3 The failure of the Hong Kong anti-trafficking regulatory regime: *ZN v Secretary of Justice*

The problem, from the perspective of human trafficking, however, is that whilst these prohibitions may indeed take effect in Hong Kong law, they do nothing to ensure that those liable for forcing another into servitude, such as in domestic forced labour situations, are also capable of being recognised as traffickers. The law, in prohibiting forced labour without reference to its potential as a trafficking activity, thereby fails to align with the Palermo Protocol’s understanding of trafficking under which the exploitation for the purposes of forced labour or domestic servitude could constitute “trafficking” as long as the other two elements (that of control, harbouring, transfer, or receipt of persons, and of there being some force, threat, deception, or coercion) have been present. The consequences of this, again, are that the authorities in Hong Kong are denied the ability to identify trafficking, the perpetrators of trafficking (and subsequently the criminal gangs and enterprises responsible for organising this sort of trafficking), and to break such networks.

More practically, there is a hypothetical loophole which arises here in which a person might be required to “receive” another person, who has been “trafficked” under the Palermo Convention, and to hold such a person until they are transferred subsequently to their new place of forced labour. Even if the authorities in such a situation were alerted to the circumstances, because the person detaining or holding the trafficked person had not themselves forced the other into forced labour, they would be unable to be prosecuted for such an offence unless it could be shown that the purpose of the exploitation was specifically prostitution and that the individual had been trafficked into or out of Hong Kong for such a purpose. In other words, the law on forced labour in Hong Kong fulfils only partially, or only one, of the three elements of the Palermo Protocol’s definition in the form of exploitation.

The inadequacy, therefore, of relying on a specific forced labour prohibition which is entirely distinct from and separate from the law on trafficking is clear. This was seen with some clarity in the Hong Kong Court of Final Appeal’s decision in *ZN Secretary for Justice and Others* [2019] HKCFA 53. In *ZN*, a Pakistani national had complained to the authorities that he had been falsely promised a lucrative job working in Hong Kong and, upon his arrival, had his passport confiscated and was forced into servitude performing unpaid domestic labour. The complainant’s wages were continuously withheld for a period of years at a time, and he was forced to remain and sleep in his employer’s office and to work 7 days a week. Given that the individual had not been put to sexually exploitive purposes, the authorities were, at first, unclear on how to process or protect the complainant (Segate, 2020). After being moved around several different departments, such as the Labour Department, the Police, and the Immigration Department. The complainant eventually brought an action against the Secretary of Justice, asserting that the state had failed to fulfil its obligations under the BOR to ensure that he was protected from being trafficked and asserting that if the BOR in Article 4 did not contain a prohibition against human trafficking for the purposes of exploitation other than for sexual exploitation, then the State was under an obligation to introduce specific legislation in order to prohibit trafficking of such a nature.

As Huang (2021) suggests, the HKCFAR in the *ZN* case therefore had a real opportunity to require an amendment of the law here, being invited by the complainant to declare that the legislature was not fulfilling its obligations under the ICCPR by failing to prohibit trafficking for the purposes of forced labour, thereby side-stepping the fact that Hong Kong remains a non-signatory to the Palermo Convention itself. This was made possible, according to submissions put forward by the plaintiff, by the fact that the *travaux preparatoires* of the ICCPR in Article 8 (which prohibits servitude, slavery, and forced labour as noted) included notes suggesting that “servitude” had to be interpreted widely as including any sort of servitude. The point, according to the plaintiff in *ZN*, of such a submission was to show that Article 8 of the ICCPR (and thus, reading down, Article 4 of the BOR itself in Hong Kong law) could be read as being a prohibition not just to the substantive conduct of forcing another to work for that person, but also to the processes by which such conduct takes place, in the form of trafficking. In other words, the plaintiff in *ZN* identified that the first of the three elements constituting “trafficking” in the form of the act or process of trafficking (the harbouring, receipt, transfer, etc. of person) was itself something which could be prohibited under Article 8’s prohibition of forced labour or servitude.

Ultimately, however, the court refused to make such a declaration. The HKCFAR declared that Article 4 of the BOR did not in fact prohibit “trafficking” and instead only prohibited forced labour or servitude. The court, in doing so, adopted a strict, textual approach to interpretation of the ICCPR and the BOR itself, declaring that Article 31(1) of the Vienna Convention on the Law of Treaties 1969 (the United Nations Convention on the Law of Treaties 1969) (the VCLT) required such an approach to be taken, so that the words of the treaties be given their ordinary meaning, in light of the context, object, and purpose of the treaty itself. Accordingly, the court held that even if human trafficking could be identified as being a process (a means) of constituting “slavery and the slave trade in all its forms,” the specific prohibitions in Article 4(1) of the BOR, Article 4(2), and 4(3)(a) constituted prohibitions of substantive conduct, not processes. Thus, the prohibition on servitude could only be considered a prohibition of the state of being held in servitude, not of the “anterior process by which a person might be brought to that status.”

The logic of the Permanent Judge of the Hong Kong Court of Final Appeal, Fok PJ here in *ZN*, as set out in paragraph 44 of the *ZN* judgement is difficult to disagree with; courts must interpret treaty provisions and legislative provisions as they are set out according to their ordinary meaning within the context and purpose of that legislation, trusting the legislature to have chosen the words and approach which they intended to apply. It is not the place of a court to replace the words or meaning of a treaty with the court’s own understanding of what the treaty ought to read, in an ideal world, a point identified by Fok P. J. at paragraph 29, when reference was made to Lord Bingham’s judgement in the UK’s House of Lord’s decision in *R (European Roma Rights) v Prague Immigration Officer* [2005] 2 AC 1 (HL) [Bingham L. at (18)–(19)]. The natural consequences of this logic, however, subsequently meant that the court was also unable to declare that the legislature in Hong Kong was under an obligation to introduce a specific piece of trafficking legislation; the state had failed to sign and ratify the Palermo Protocol, and it would not be possible for the judiciary to ignore that and impose the Convention’s requirements irrespective of such a decision by the legislature.

In conclusion, the failure by the legislature in Hong Kong to introduce a specific, anti-human trafficking law which prohibits trafficking according to the three elements set out in Article 3 of the Palermo Protocol on the basis of the process of trafficking, the act of coercion of threat, for the purposes of exploitation leaves the law in Hong Kong in a difficult place, unable to provide assistance to trafficking victims and unable to identify and bring to justice the perpetrators of such acts. For Hong Kong, there is concern that serious consequences may stem from such a failure; as Huang (2021) writes, the US State Department, which has been monitoring Hong Kong’s compliance with anti-trafficking rules, downgraded Hong Kong’s status to the “Tier 2 Watch List” (the second lowest tier), and any further downgrade would be required under US law to be accompanied by trade sanctions being imposed on the region. This is a serious threat, and the legislature in Hong Kong appears prescient of such concerns. The Steering Committee to Tackle Trafficking in Persons and to Enhance Protection of Foreign Domestic Helpers, for example, has reiterated the state’s intent to pass a Modern Slavery Act of its own, and the “Action Plan to Tackle Trafficking in Persons and to Enhance Protection of Foreign Domestic Helpers in Hong Kong of 2018” has now seen the US State Department move Hong Kong back up to “Tier 2” in 2019, removing for the time being the imminent threat of sanctions. However, as Huang (2021) goes onto note, there remain concerns that these steps are little more than efforts to appease the international community. The legislature has had a Modern Slavery Bill on its books since 2017, and yet has continued to take no steps to pass this bill into law, blaming legislative time-table interruptions as a result of the COVID-19 pandemic, for example, for such a failure (Glavine, 2015).

If the Hong Kong legislature is indeed serious about addressing the deficiencies in its legislative framework surrounding human trafficking, the question arises: what form should such a model of legislation take? To answer this, it is possible to examine the approaches taken in Europe, specifically within the EU and the UK, to identify lessons that Hong Kong can learn.

## 3 Lessons from Europe and recommendations for Hong Kong

### 3.1 The EU and Council of Europe’s approach to combatting human trafficking

In somewhat stark contrast to the failure of the legislative council of Hong Kong to take concrete action to prevent and combat human trafficking in a manner which conforms to the requirements of the Palermo Protocol, other states around the world have been much more enthusiastic (Gebrewold et al., 2017). In the EU, there has been a significant emphasis on regulatory frameworks surrounding the suppression of organised crime through anti-money laundering frameworks, for example, in recognition of the societal harm which such organised crime can cause and partly because of the threat of terrorism faced by several states in Western Europe and in the UK in particular. Given that the global value of human trafficking is estimated by some to be worth $150 billion to trafficking criminal enterprises globally every year, there is an obvious incentive to seek to restrict and combat human trafficking (Gebrewold et al., 2017, p. 2).

It should be noted at this point that this is not, however, something restricted solely to the EU as an institution; the Council of Europe, a treaty organisation with 46 Member States, including the EU’s own 27 Member States, but also including, amongst others, the UK, Turkey, Switzerland, and Norway, for example, has developed its own anti-human trafficking agreement. This is set out in the 2005 Council of Europe Convention on Action against Trafficking in Human Beings. The Council of Europe’s Convention again defines, in Article 4(a), trafficking according to the three elements of trafficking recognised in the Palermo Protocol of acts, means, and purpose. Article 4(a) thus provides that “trafficking” occurs whenever there is the “recruitment, transport, transfer, harbouring or receipt of persons” just as the text of Article 3 of the Palermo Protocol provides.

Furthermore, Article 4(a) explains that this act of trafficking is such if it is constituted by the means of “threat or use of force or other forms of coercion, of abduction, of fraud, of deception, of the abuse of power or position of vulnerability or of the giving or receiving of payments” all of which are intended to achieve the consent of the person to being trafficked. The purposive element in Article 4(a) is the purpose of “exploitation,” with it being specifically provided, as is the case under the Palermo Protocol, that exploitation is not restricted (as is the case in Hong Kong in order for trafficking to be recognised) to sexual exploitation such as prostitution but also includes, as a minimum, forced labour or services, slavery, or other similar practices.

The Council of Europe’s signatory states are required under the Convention to establish national measures not only to prevent trafficking in human beings in their own jurisdiction [under Article 5(2) but also, under Article 10(1) and (2)], to ensure that steps are taken to ensure the proper identification of victims of trafficking, and to ensure the protection of such persons. Again, this is something which the law in Hong Kong falls short on, with it being said by several, such as Chan (2014), that one of the great difficulties for investigators in Hong Kong results from the fact that trafficking victims other than those who might be at risk of sexual exploitation, such as by being coerced or forced into prostitution, are not readily identified by the authorities. The reason for this, in Hong Kong, is largely due to the fact that there is no holistic top-down approach to combatting trafficking being required by law (even though the 2018 Action Plan as noted above does contain elements of such a process, this lacks legal force). The Hong Kong Justice Centre, for example, explains that in order to properly identify those at risk of trafficking both cross-border co-operation with other law enforcement agencies and international organisations is required, along with co-operation and communication between various departments of the state, such as border and immigration officials, the police, and others such as NGOs who have special expertise in identifying and assessing risk factors for forced labour or servitude (Justice Centre for Hong Kong, 2014).

Without a legal definition and prohibition of trafficking similar to that adopted under the Palermo Protocol or under the Council of Europe’s Anti-Trafficking Convention, the authorities in Hong Kong lack a set of protocols and guidelines and a legal framework through which to begin the identification and processing of these victims. This was shown to be the case clearly in the *ZN* case, where the trafficked complainant was passed back and forth between several different departments and agencies for some time, with it being unclear which department ought to process him, or whether any criminal offence had been committed against him.

The position in Hong Kong here in respect to lacking a holistic and unified framework or strategy to combat human trafficking can also be contrasted with that of the EU, which has recently adopted a new “EU Strategy on Combatting Trafficking in Human Beings 2021–2025”. As the European Commission (2021) has made clear, this is part of the EU’s attempts to support and facilitate the effectiveness of the Palermo Protocol, to which the EU is itself a signatory, along with the EU’s own Member States. This allows the EU to adopt specific “action plans” to counter prescient threats and avenues through which trafficking is perceived to be taking place, such as across the Mediterranean Sea, for example, where groups of desperate migrants and refugees are often trafficked by criminal gangs and organisations.

The core legal elements of the EU’s anti-trafficking regulatory framework, however, are contained in the EU’s Directive 2011/36/EU (the Trafficking Directive), which replaced the Council’s former Framework Decision implementing the Palermo Protocol. The Directive requires all Member States to “take the necessary measures to ensure” that certain prohibited acts are punishable by law. These acts, set out in Article 2(1), establish the Palermo Protocol’s definition of “trafficking,” which is based on the three elements of the “act,” the “means” and the “purpose” of trafficking. The wording of Article 2(1) and the definition of trafficking in EU law are in fact taken verbatim from Article 3(a) of the Palermo Protocol, and thus the Protocol is effectively incorporated into EU law. As is the case under both the Palermo Protocol and the Council of Europe’s Convention, “exploitation” is also defined in Article 2(3) of the EU’s Trafficking Directive as including “as a minimum” the exploitation of prostitution or sexual exploitation, as well as forced labour or other services, slavery, practices similar to slavery, or servitude or the removal of organs. However, the EU’s Directive goes further still, specifically including a number of other forms of behaviour which are regarded as being, at a minimum, exploitative, such as the exploitation of other’s begging behaviours, the exploitation of criminal behaviours (Article 2(3) Directive 2011/36/EU). Thus, there has been some recognition from the EU as to specific risk factors which might arise within the Member States and which could otherwise potentially pose difficulties to the courts of the Member States and to the Court of Justice of the European Union (CJEU) if these behaviours were not expressly provided to be “exploitation.”

One further important element of the EU’s Trafficking Directive is that set out in Article 5, which makes clear that it is not only natural persons who can be made liable under the Directive. Instead, it is required that Member States take the necessary measures to ensure that legal persons (corporate entities) operating within their territory are also able to be liable for the offences, such as those of trafficking, set out in Article 2. Finally, under Articles 11 and 12 of the Directive, provision is made to require Member States to ensure that they provide assistance and support to the victims of trafficking, again in line with the Palermo Protocol’s requirements, and to help protect vulnerable individuals and trafficking victims from criminal enterprises so that they can give evidence effectively, such as by restricting unnecessary interviews, ensuring use of technologies and video links, for example, for giving of evidence, and so on. All of this is evidence of a well-thought-out, holistic strategy to combat trafficking, which focusses not only on an accepted international definition of trafficking and all of its elements, but also on ensuring that trafficking victims, and the perpetrators of such acts, can be identified, protected, or prosecuted effectively, as the case may be.

### 3.2 The UK’s anti-human trafficking endeavours

The UK remains a Member State of the Council of Europe and is therefore bound by the provisions of the Council of Europe’s Anti-Trafficking Convention. The UK was, until 2020, a Member State of the European Union, and before it exited the EU, a Member State had an “opt-out” as part of its membership status at the time, which meant that it would not automatically be required to transpose the directive. The UK Government, in 2011, however, did agree to “opt-in” to the Directive, meaning that the UK indicated an intent to introduce legislation to transpose the directive. This legislation eventually took the form of the Modern Slavery Act 2015. The Modern Slavery Act, however, far from being a provision which replicates the approach taken under the Council of Europe’s Convention or under the EU’s Trafficking Directive, takes a rather novel approach to the question of “trafficking” and its definition. This is because discourse in the UK since the launch in 2014 of the UK’s “Modern Slavery Strategy” has identified “trafficking” as a subset of a form of behaviours known as “modern slavery,” with trafficking therefore being a behaviour which falls under the general “umbrella” of modern slavery, along with forced labour, servitude, and slavery. This approach risks conflating the ideas of forced labour and slavery with the exploitative purpose of trafficking as defined under the Palermo Protocol and the Council of Europe’s Convention. The UK’s strategy and regulatory framework here have been criticised as a result by some, such as Broad (2019), with it being suggested that the legislation is rather unwieldy, overly complex, albeit, ultimately, compliant with the Palermo Protocol (and therefore at the time the EU’s own 2011 Trafficking Directive) as well as the Council of Europe’s Convention.

This can be seen by considering the way in which s2 of the Modern Slavery Act 2015, which creates the specific offence of “trafficking,” operates in the UK. Here, s2(1) provides that a person commits the offence of “trafficking” if they “arrange or facilitate” the “travel” of another person with a view to their being exploited. Thus, two elements of trafficking in the form of the “act” of trafficking (in UK law, performed by the facilitation or arrangement of “travel”), and the third element of trafficking, in the form of the purpose of such travel being “exploitation,” are present. The second element set out in the Palermo Convention’s definition of trafficking, that of there being a threat or use of force, coercion, deception, fraud, abuse of power, and so on, is missing from section 2 of the Act, and it is expressly provided in s2(2) that the victim’s free consent is irrelevant, as long as the individual is indeed being moved with the intent of their being “exploited.” However, section 3 of the Act goes on to explain that “exploitation” for these purposes can include any situation, not only where the person travelling is being required to perform any act which might constitute a criminal offence (including an offence under the Human Tissue Act 2004 (Part 1 Human Tissue Act 2004), which prohibits the commercial dealing of human organs), but also where the victim is subjected to force, threats of force, or deception designed to induce them to provide services of any kind or to provide benefits of any kind to another or to enable another to do so. Thus, in actuality, all three elements of the “trafficking” definition set out in the Palermo Protocol, in the “act,” the “means,” and the “purpose” of trafficking are present, albeit in a rather unwieldy and complex legislative text.

Despite this, there remain criticisms of the UK’s regulatory approach. As Cooper et al. (2017) write, there have been relatively few successful prosecutions for trafficking offences which have taken place in the UK since the coming into force of the Modern Slavery Act 2015. Furthermore, research by these authors suggests that many traffickers continue to engage in their trafficking acts motivated, amongst other things, by a belief in a low level of risk of detection or conviction. This is indicative of criticism of the UK’s overall approach to combatting trafficking, which is argued by Broad (2019) as resulting from the fact that the UK’s overall strategy to combat trafficking is only “moderately structured.” Others, such as the Anti-Trafficking Monitoring Group (Anti-Trafficking Monitoring Group, 2018), have argued that whilst the UK has indeed attempted to comply with its Convention obligations by, *inter alia*, establishing independent bodies to monitor anti-trafficking efforts (such as the Gangmasters and Labour Abuse Authority or GLAA, the Joint Slavery and Trafficking Analysis Centre or JSTAC, or the Independent Anti-Slavery Commissioner or IASC), in line with Article 29 of the Council of Europe’s Convention, these efforts have only been moderately successful. For the Anti-Trafficking Monitoring Group, however, these failures are not due to legislative weakness but rather to concerns over funding for these authorities and monitoring groups and a lack of independence of the IASC, or Special Rapporteur, who at present is not answerable directly to Parliament and who lacks the authority to require access to data held by the Police or National Crime Agency, for example. These are structural weaknesses of the framework of anti-trafficking regulation in force and are not, in themselves, weaknesses of the UK’s legislative framework *per se*.

### 3.3 Hong Kong’s Modern Slavery Bill assessed against the UK and European model

In making recommendations for Hong Kong here, in terms of its legislative framework, it is certainly possible to suggest that adoption of a new legislative framework through the Modern Slavery Bill of 2017 would help to improve anti-trafficking efforts in Hong Kong. The draft bill, presented to the legislature of Hong Kong, was based on the UK’s Modern Slavery Act 2015. The bill would create a new Part XIV to the Crimes Ordinance (Cap 200), which would include within it a new section 163 creating a specific offence of “human trafficking” removed from the current Hong Kong paradigm which requires transit of a person into or out of Hong Kong specifically for the purposes of prostitution or sexual exploitation. Instead, the new section 163 would create an offence for trafficking, which would occur whenever a person “arranged” or “facilitates” the travel of another with a view to their being exploited, with travel, as it is under s2 of the UK’s Modern Slavery Act 2015, being fulfilled not only by international transit but also by the “travelling within” the region of Hong Kong or indeed any other country. Additionally, exploitation, as is the case under the UK law here, would be explained in s164(5) as including the procurement of any services by that person achieved by force, threats, coercion, or deception.

In short, the approach taken in the UK would be replicated by Hong Kong, so that for the first time, there would be a Hong Kong trafficking offence which fulfilled the definition of “trafficking” set out in the Palermo Protocol. All three elements of “act” of “means” and of “purpose” would be recognised by such a law, and this is therefore to be recommended here. However, suggesting that this alone would be sufficient to prevent or protect individuals from the risk of trafficking would appear naïve in light of the UK experience and that of Europe more generally. It is clear that the key concern for the authorities here is to find some way of ensuring that trafficking victims and perpetrators can be identified whilst engaging in the act where possible. The Palermo Protocol and the approach taken in the Council of Europe’s Anti-Trafficking Convention recognise this and place emphasis on the signatories adopting a range of protocols and policies designed to ensure co-operation both internationally between signatories, and domestically, between various agencies of the state and NGOs so that a consistent and identifiable single strategy on anti-trafficking is capable of being adopted by the state.

### 3.4 Human Rights Law in Europe: the European Convention on Human Rights and the impact of the European Court of Human Rights

Another element of the more holistic approach taken in Europe to anti-trafficking efforts, compared to that in Hong Kong, is facilitated by the European Convention on Human Rights (ECHR) 1950. With the exception of the Russian Federation, which is no longer a member of the Council of Europe, all Council members are signatories to the ECHR. The Convention (or ECHR) was the “first comprehensive treaty in the world” of its type (Steiner and Alston, 2008, p. 786) as it was the first binding, and mandatory international human rights treaty which imposed specific obligations on its signatories to protect the rights of those within their control or jurisdiction. The ICCPR, which was itself based on the Universal Declaration of Human Rights, followed this in other parts of the world and has been influential in Hong Kong in the development of Hong Kong’s BOR, as noted earlier. As the ICCPR does in Article 8, Article 4 of the ECHR prohibits slavery and forced labour, with Article 4.1. providing that no one shall be held in such condition (that of slavery or servitude), whilst Article 4.2. prohibits forced labour or compulsory labour, subject, in Article 4.3. to a number of exceptions such as those also seen in the ICCPR and in Article 4 of the Hong Kong BOR, which are work imposed as part of detention for criminal sentences, military service, or in the event of natural disasters, for example.

Being drafted as the ECHR was, before the advent of modern understandings of the definition of “trafficking,” there is nothing expressly provided for in the ECHR or in Article 4 itself in respect of the phenomenon of “trafficking” itself. Trafficking is, however, suggested by the European Court of Human Rights in *Rantsev v Cyprus and Russia* [*Rantsev v Cyprus and Russia* (Application No. 25965/04 [2010] 51 EHRR 1)] and in *M and others v Italy and Bulgaria* [*M and others v Italy and Bulgaria* (Application No. 40020/03 [2012] ECHR)] to be a sort of activity which amounts, in and of itself, to “inhuman and degrading treatment” contrary to Article 3 of the Convention. This is important. As the court in *M and others* held at paragraph 106, this in turn creates a “positive” duty upon the State to investigate effectively and to offer protection to victims of trafficking where it is suspected or feared that such trafficking might take place. In making such a ruling, there is a clear lesson which could be learned and which might be applicable for Hong Kong and for the HKCFAR in the *ZN* case, which is that the failure of any express “trafficking” provision being set out in law ought not necessarily to have precluded a finding that the state was under a positive obligation to take steps to investigate or prevent such trafficking occurring. In this respect, at least, the European Court of Human Rights can be seen to have adopted a relatively novel and pioneering approach to interpretation of “trafficking.”

In other words, the European Court of Human Rights has effectively addressed the challenge posed by the ECHR’s lack of a definition or explicit prohibition of “trafficking” as a distinct act. This was notably demonstrated in the *Rantsev* case, where the Court employed a contextual interpretation of international law, particularly referencing instruments like the Palermo Protocol. Through this approach, the ECHR recognised that the Palermo Protocol’s definition of trafficking—comprising three elements: the act, the means, and the purpose of exploitation—parallels the concept of modern slavery. The primary focus is on the exploitation of human beings, which inherently disregards individual autonomy. Since the ECHR prohibits slavery under Article 4, which includes the ownership or exercise of ownership-like rights over another person, the Court in *Rantsev* concluded that trafficking, as an exploitation based on the exercise of rights over another individual, can be encompassed within the broad understanding of modern slavery.

The *Rantsev* case is important in which it highlights a different theoretical and scientific method by which a court could use established definitions such as those of trafficking, of slavery, to ensure that there is an obligation on the state to take steps to prevent trafficking from a human rights perspective, even if no express obligation to protect against trafficking as defined in the human rights law governing the state’s obligations in statute here is found (Stoyanova, 2016, p. 163).

The difficulty for the European Court of Human Rights in *Rantsev* was to be found in the definition contained in Article 4(1) which provides that:

“No one shall be held in slavery or servitude.”

Article 4(2), meanwhile, provides:

“No one shall be required to perform forced or compulsory labour.”

These are the basic prohibitions set out in Article 4 and are subsequently subjected to a number of exceptions and exemptions, such as allowances being made to allow states to take action during times of war or catastrophe or for states which require national service to be performed, for example, by their citizens.

As can be seen from the above, there is no explicit mention of “trafficking” within the scope of Article 4. Moreover, both terms, slavery and servitude, have their own discrete definitions (Egan, 2015). The definition of “slavery” was accepted by the court in *Siliadin v France* (Application No. 73316/01 [2005] 43 EHRR 16) as being that set out in the Slavery Convention 1926, which itself defines slavery as being “the status or condition over a person whom any or all powers attaching to the right of ownership are exercised” (Cullen, 2006). Servitude, meanwhile, was accepted by the ECHR in cases such as *Seguin v France* (Application No. 42400/98 [2002] ECHR 420), as being constituted by an obligation to provide one’s services, that is, imposed as a result of coercion.

In short, both concepts share common elements with the offence of trafficking as defined under the Palermo Protocol; they are built on the assumption of a person’s free will being made by another in order to exploit the other. There are, however, fundamental differences between the three, which is ultimately why they remain three separate offences.

In servitude, unlike slavery, it is not, for example, necessary for a person to be “owned” by another or to have any suggestion that the other has ownership rights over the other; thus, whilst a slave will always be kept in servitude by their owner, the person kept in servitude may not be a slave. Similarly, a person who is trafficked under the definition provided for in the Palermo Protocol is someone who is trafficked for the “purposes” of exploitation; whether they are actually exploited in the end is irrelevant; it is the motive or purpose which is important for the definition of trafficking (Jansson, 2015, p. 105). There is not necessarily, however, any requirement that a trafficked person have their rights of personhood “owned” by another, nor that they actually be kept in servitude. Instead, as has been seen at length, in order to be “trafficked” a person has to have been recruited, transported, transferred, harboured, or received by another, through the threat of coercion or fraud, for those purposes. There is in fact a real danger which might be created by conflation of these terms, as Allain (2010, p. 553) argues, which would be that the distinct nature of trafficking as a standalone offence might be defeated if courts sought to impose some other obligations too upon the defendant, such as that they actually exploited the other (rather than merely having the motive to do so), or that they held the other as a slave. The European Court of Human Rights in *CN and N v France* (Application No. 67724/09) unreported Judgment of 11 October 2012 has acknowledged that there is, and ought, to be a distinction between servitude and slavery maintained under Article 4 (Scarpa, 2008).

This was the dilemma for the ECHR in the *Rantsev* case; slavery and servitude are expressly mentioned as being prohibited Article 4, and there is a positive right not to be kept a slave or in servitude, which the state is under an obligation to protect. No mention is made of any such distinct right to avoid being “trafficked,” however. If seen from the perspective of constituting specific criminal offences (rather than rights, and rather than the obligation trigged upon a state to take steps to protect individuals’ rights here), then it might also be said that trafficking, slavery, and servitude are three offences which share the same *nexus*, which is the fact that the victims’ autonomy is interfered with for the purposes of exploitation of them (Knight, 2023). It was the ECHR in *Rantsev* acknowledged when extending the scope of Article 4’s prohibition on slavery and servitude to cover, in fact, exploitation, so that it was found that a person has a right to not be “trafficked,” and furthermore, that the state is under a positive obligation to prevent that (Duffy, 2016).

The decision in *Rantsev*, and further decisions in cases such as *LE v Greece* [*LE v Greece* (Application No. 71545/12) unreported 21 January 2016 (ECHR)], in which it was again confirmed that a positive obligation exists upon the state to protect against trafficking (and thus that the state is required to take certain positive steps to determine whether a person is or has been trafficked, for example), thereby increasing, at least from one perspective, the scope of protection which ought to attach to people at risk of being trafficked. This is the alternative approach which the Hong Kong Court in *ZN* could have adopted here.

What can then be said of the concerns raised by Allain here? Allain’s criticism of the *Rantsev* decision primarily focusses on the fact that by conflating slavery, servitude, and trafficking, the ECtHR in *Rantsev* largely ignores the formulaic definition, and two of the three elements of “trafficking” which have been identified at length in this article (the acts, the means, and the purpose). The court, for example, ignores the fact that in order to be trafficked, a person is required to be “received,” “harboured,” or “transferred.” Allain argues that by determining, on the one hand, that the scope of Article 4 ought to be extended beyond its textual scope to cover trafficking, whilst, on the other, failing to determine specifically under which provision (slavery or servitude) trafficking falls, shows a misunderstanding of the “standing or substance” of Article 4 (Allain, 2010, p. 555). In other words, the court fails to understand the reason why slavery, servitude, and trafficking ought to be seen as being three different concepts (Allain, 2009).

This can be seen to have some value as a critique. If the court demands that a person be kept in a condition “akin” to slavery, then there may be a very real risk that the threshold for the offence of trafficking to be committed is raised by the *Rantsev* decision. For Allain (2010), if one replaces (as the court in *Rantsev* effectively does) the phrase “the exercise of powers attaching to the right of ownership” for the simple word “slavery,” then the court in *Rantsev* suggests that the offence of trafficking is one which “by its very nature and aim of exploitation, is based on slavery.” This, however, is not necessarily true; slavery requires the rights of ownership of a person to be exercised by coercion or force; what of a person who is trafficked by deception to give their organs willingly to another, thinking it was for their relative, for example, when it was really for the purposes of commercial exploitation? (Selimi, 2016). In such case, it would be difficult to say that “slavery” was found, or “servitude” as no labour is carried out by the victim (Allain, 2015), and nor are any rights of ownership ever really exercised over the other through force; it is only a case of deception, which is turned into a trafficking offence by the “act” of trafficking (the “recruitment, transfer, harbouring, etc.”; Todres, 2013). It is therefore arguable that the *Rantsev* judgement creates some degree of danger of confusing and undermining the clear trafficking definition. Doing so, however, in pursuit of ensuring that states bear a positive duty to protect individuals under human rights law from being trafficked may be worth this risk (Piortorwice, 2012).

The failure to recognise the exploitative nature of trafficking in the *ZN* case as a violation of the Hong Kong BOR and its prohibition on slavery may partly stem from the Hong Kong legislature’s failure to align the human trafficking definition with the Palermo Protocol. Without such alignment, the existing definition of human trafficking in Hong Kong law remains overly strict and confined to sexual exploitation. This narrow scope likely made it difficult for the court to interpret trafficking broadly, as being akin to slavery or forced labour.

This understanding of trafficking is one which respects the harm and interference with the dignity of the victim which trafficking causes, and the HKCFAR in the *ZN* case could quite easily have held that trafficking, resulting in “inhuman or degrading” treatment under Article 7 of the ICCPR, did indeed impose positive obligations on the state to adopt policies and laws designed to prevent such trafficking from taking place in the first case. When seen in light of the European Court of Human Rights’ jurisprudence here, the *ZN* case does appear to represent a missed opportunity to spur the Hong Kong legislature into action.

## 4 Conclusion

The failure of the Hong Kong legislature to pass the Modern Slavery Bill of 2017 now renders Hong Kong something of an international outlier in its anti-human trafficking efforts. In the European Union, the EU’s Member States, in transposing Directive 2011/36/EU, and in being signatories to the Council of Europe’s Anti-Trafficking Convention, have all adopted a model of anti-trafficking law which is built on a common understanding and definition of “trafficking.” This definition identifies trafficking as being performed by the carrying out of an action of recruitment, harbouring, transfer, or transit of another person, which is procured by a means, by way of force, threat, deception, or fraud, and which is intended to result in the exploitation of the victim. Hong Kong’s law, which prohibits only the trafficking of persons for the purposes of sexual exploitation or prostitution, fails to fulfil the minimum accepted international standard here. As the law in Hong Kong only prohibits sexual exploitation as a “purpose” instead of a wider understanding of the reasons why people are trafficked, those forced or deceived into domestic servitude or other forms of exploitation receive no recognition, nor identification in Hong Kong, as victims of trafficking.

To an extent, this appears intentional. There is a concern, as was put forward in submissions by counsel for the Secretary of Justice in the *ZN* case, that recognising other forms of exploitation, as the Palermo Protocol demands, is liable to subsequently give rise to a number of other obligations to protect those individuals, to provide them housing and opportunities for education and training, for example. This is true. However, it is astonishing that the Hong Kong legislature fails to acknowledge the seriousness of the harm that is done to victims of trafficking when this is performed not only for the purposes of their sexual exploitation but for other reasons too. Every State that is a signatory of the Palermo Protocol, or indeed, the Council of Europe’s Convention, acknowledges this and has agreed that it is implicitly necessary for the state to bear the cost of protecting these vulnerable individuals from harm.

Whilst the state in *ZN* argued that this is not necessary, as the BOR already protects individuals from servitude, forced labour, or slavery, this is not strictly accurate because it ignores the necessity for a holistic and consistent anti-trafficking strategy in order to properly ensure the identification of trafficking, to identify and protect its victims, and to identify and prosecute its perpetrators. This does not require the Palermo Protocol to be signed in order to be achieved; the legislature could do so by bringing domestic law in Hong Kong into alignment with the Protocol by passing into force the Modern Slavery Bill of 2017 and by subsequently developing and creating the institutions and agencies necessary to properly monitor trafficking and to engage with NGOs and other states and their own agencies and crime-fighting bodies more effectively. Whilst the legislature in Hong Kong fails to take the problem of trafficking seriously and enact a dedicated anti-trafficking law, the development of the necessary institutions and agencies to effectively monitor and prevent trafficking is hindered. Without a specific, defined, and widely understood criminal offence. These different agencies cannot effectively coordinate and focus their efforts. In conclusion, this article recommends that the Hong Kong legislature learn from the efforts taken to combat human trafficking in Europe by either ratifying the Palermo Protocol, or, by passing into law the Modern Slavery Bill of 2017 at the earliest opportunity.
